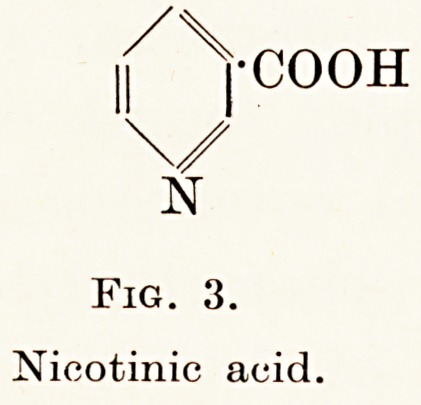# The Vitamin B Complex

**Published:** 1938

**Authors:** H. M. Sinclair

**Affiliations:** Department of Biochemistry, University of Oxford; Fellow and Tutor, Magdalen College, Oxford


### THE VITAMIN B COMPLEX.

BY

H. M. Sinclair, M.A., B.Sc., B.M., B.Ch.,

*Department of Biochemistry, University of Oxford ;*
*Fellow and Tutor, Magdalen College, Oxford.*

---

### Introduction.

Although scurvy, beriberi, rickets and pellagra have been known for centuries, the discovery of diseases due to vitamin deficiencies dates only from the beginning of this century. It is true that Lind (1753) made some admirable observations on scurvy ; that Stark in 1769 twice induced the disease in himself by careful dietetic experiments (as a result of which he died) ; and that Michael made excellent attempts to isolate the antiscorbutic principle early in the eighteenth century. Further, Lunin (1881) clearly recognized that small quantities of unknown substances were essential to life, and Pekelharing (1905) demonstrated the existence of diseases due to dietary deficiency. Yet it was Hopkins (1912) who first realized the full significance of vitamins. Since then their number has increased with such rapidity that it is a year or two since the discovery of vitamin T was claimed ; vitamin B has been split into some dozen possible factors, and about two new papers on vitamins B and C are published each day of the year.

Much of this work will not stand a critical analysis, and it is obvious that the clinician cannot hope to be fully conversant with the scientific work being done in this field. It is the object of this paper briefly to summarize recent work, done mainly upon animals, on the vitamin B complex, and as far as possible to indicate applications of this work.

The term " water-soluble vitamin B " was originally used for the antineuritic vitamin discovered by Eijkman in 1897. During the last twelve years it has been shown that this factor is multiple. Vitamin $B_2$ was distinguished in 1926 as a factor which prevented pellagra and was more heat-stable than vitamin $B_1$. Vitamins $B_3$, $B_4$, $B_5$ and $B_6$ were later recognized; all are water-soluble and present in fresh yeast, and all are necessary for the nutrition of pigeons or rats. Confusion then arose. Rats fed on a diet deficient in vitamin $B_2$ failed to grow and developed a dermatitis, and this was believed to be analogous to pellagra. When the fluorescent compounds called flavins came into prominence in 1933, it was found that one of these (riboflavin) could be isolated from preparations of vitamin $B_2$ and would promote growth in rats adequately provided with vitamins $B_1$, $B_4$ and $B_6$. But pure riboflavin was found to have no effect upon this dermatitis of rats or upon human pellagra; it was obvious, therefore, that vitamin $B_2$ must consist of more than one factor. In 1935 it was shown that the factor which prevented rat dermatitis was vitamin $B_6$, and so for the rat vitamin $B_2$ consisted of riboflavin and vitamin $B_6$. Neither of these components, either alone or in combination, cured human pellagra, and therefore the original " vitamin $B_2$ " (which, of course, had never been isolated pure) consisted of at least three factors: riboflavin, vitamin $B_6$, and the P-P (pellagra-preventive) factor, now believed to be nicotinic acid or a derivative (see p. 185). These factors are all adsorbed on fuller's earth, but there is a fourth factor necessary for the growth of rats and chicks which is not adsorbed (and called on this account the " filtrate factor "). These four factors make up the vitamin $B_2$ complex.

Vitamins $B_3$, $B_4$ and $B_5$ are not known to have any clinical significance, and other alleged constituents of the vitamin B complex (such as factor W, choline, vitamin H, and the " anti-gizzard-erosion factor for chicks " described by Bird *et al.* (1936)) need not detain us from the more detailed consideration of vitamin $B_1$ and the vitamin $B_2$ complex.

### VITAMIN $B_1$.

### *Clinical Considerations.*

More than fifty years ago Baron Takaki deduced that beriberi was of dietary origin, and, by adding more meat and vegetables to the diet of Japanese sailors and by partly replacing the customary rice by wheat and barley, greatly reduced the incidence of the disease in the Japanese navy. Despite the mass of work which has been published upon the clinical manifestations of deficiency of vitamin $B_1$, this is still a frequent cause of illness and death in such countries as China, Japan, Brazil, the Philippine Islands and some parts of the United States. Gross deficiency is undoubtedly rare in this country, but mild and subclinical states are probably fairly common.

In man symptoms of deficiency begin and progress somewhat as follows. First there is loss of appetite, with lack of energy, muscular weakness and "heaviness" of the legs; this is followed by tenderness of the calf muscles, and "burning" of the soles of the feet with loss of vibration-sense in the legs. The paræsthesiæ in the legs become more marked, the weakness increases and is perhaps accompanied by intermittent claudication, and the Achilles and patella reflexes become weak and later absent. The weakness spreads to the extensors of the toes, then of the foot and next to the extensors and flexors of the leg. Toe- and foot-drop are therefore found. At the same time there is hyperæsthesia followed by anæsthesia advancing up the leg. Atrophy of the muscles and skin occurs. The upper extremity is usually affected later. The central nervous system is rarely involved, but the memory is usually defective. Some authors consider that both Korsakov's psychosis and the toxic amblyopia of alcohol- and tobacco-addicts are due to deficiency of vitamin $B_1$.

So far as cardiovascular symptoms are concerned, dyspnœa, "palpitations" and tachycardia are found. Later the heart dilates. The arterial pressure is usually normal and the pulse pressure slightly increased. The circulation rate is increased because the peripheral small vessels are dilated; and this dilatation is the probable cause of the œdema, which may be mild or extreme. Death finally occurs from heart failure.

At autopsy very few pathological changes are usually found except the dilatation of the heart. In chronic cases there is marked degeneration of the myelin sheaths of peripheral nerves; this commences in the periphery of the long nerves and gradually extends towards the cell.

Two factors, acting either singly or in conjunction, may be responsible for the occurrence of hypovitaminosis $B_1$:

(i) Inadequate ingestion of the vitamin;

(ii) Failure to assimilate the vitamin.

Each of these will be considered in turn.

### 1. *Inadequate Ingestion.*

Cowgill[1] (1934) has shown that the body weight and the total metabolism or calories are two important variables determining the requirement of vitamin $B_1$. Beriberi is commonest among young adult males because their metabolism is higher than that of females. The breast-fed infant is next in order of susceptibility. The incidence among women is less, and among the elderly and the aged beriberi is comparatively rare. Since the metabolism is raised during pregnancy, and since it has been shown that lactating animals require three to five times more vitamin $B_1$ than normal ones, it is not surprising that women become more susceptible to beriberi during pregnancy and lactation. Several workers have suggested that the polyneuritis of pregnancy is due to deficiency of vitamin $B_1$; Strauss and McDonald (1933) cured not only the polyneuritis but also the *hyperemesis gravidarum* in many of their cases by generous doses of yeast. Authors have recently reported relief of this condition by administration of vitamin $B_1$, and have advised its regular use in pregnancy. In pregnancy it has even been used for inducing labour and afterwards for increasing the period of lactation!

Any other factor, apart from pregnancy, which increases metabolism will increase the susceptibility to deficiency of vitamin $B_1$. The literature contains numerous reports of beriberi following prolonged fevers, and this association has led some clinicians to adopt the view that beriberi is an infection and not a dietary deficiency. Polyneuritis occasionally accompanies influenza, typhoid fever, leprosy, rabies, smallpox, and chronic pulmonary tuberculosis; vitamin $B_1$ is stated to be effective in post-influenzal neuritis and in the neuritis of leprosy. One might expect to find symptoms of deficiency occasionally in cases of hyperthyroidism (although such patients usually take a liberal diet); such cases have been recorded, and Means has recently quoted Weiss's opinion that "vitamin B deficiency . . . may explain why some patients with hyperthyroidism have cardiac dilatation and cardiac symptoms."

Actual deficient intake in otherwise normal people is undoubtedly rare in this country. Those people who, for financial reasons, are on low diets, have low caloric intakes, and therefore (as Cowgill has shown) they tend not to develop polyneuritis. It is for that reason that polyneuritis is very rare in cases of *anorexia nervosa*.

"Alcoholic" polyneuritis is now believed to be due to deficiency of vitamin $B_1$ and not primarily to a "neurotoxic" effect of alcohol. Goodhart and Jolliffe[2] (1938) have finally proved this. The subjects of this form of polyneuritis take a high caloric diet in which most of the energy is supplied by alcohol; as no form of spirits contains any vitamin $B_1$, these individuals suffer a deficiency. This deficient intake may be exacerbated by the presence of achlorhydria or hypochlorhydria in about 80 per cent. of patients with the disease (Minot) since hypoacidity is alleged to prevent the assimilation of the vitamin.

Other forms of polyneuritis have been claimed to be due to deficiency of vitamin $B_1$. Vorhaus, Williams and Waterman (1935) treated one hundred unselected cases of multiple and localized neuritis of diverse ætiology with vitamin $B_1$; it was claimed that only eight failed to show improvement, forty-four being cured and forty-eight improved. Toxic forms due to arsenic, lead, thallium, mercury, and nicotine have all been claimed to be improved by treatment with vitamin $B_1$. Such claims seem extravagant, especially in regard to heavy metals such as lead, which produce a polyneuritis clinically different from that caused by deficiency of vitamin $B_1$. Good effects of vitamin therapy have been reported by several authors in various forms of neuritis (localized, acute, post-diphtheritic, etc.); improvement has been claimed in sciatica and even in disseminated sclerosis. These claims are extravagant and fanciful. All that can be said is that deficiency of vitamin $B_1$ undoubtedly causes true nutritional polyneuritis, "alcoholic" polyneuritis and possibly polyneuritis of pregnancy and of diabetes (gastrogenous polyneuritis will be treated below).

2. *Failure to assimilate vitamin $B_1$.*

This may be due to (i) failure to absorb the vitamin (ii) destruction in the gut, or possibly (iii) failure to phosphorylate it.

Many cases of deficiency of vitamin $B_1$ have achlorhydria, although this is not caused by the vitamin deficiency. It has been suggested by many workers that cases of pernicious anæmia might be partially deficient, and that subacute combined degeneration is due to chronic deficiency of vitamin $B_1$. Analyses of blood which I have done during the last two years lend no support to this view. However, I have found low values in some cases of idiopathic hypochromic anæmia, which is, of course, sometimes caused by nutritional deficiency of iron, sometimes by failure to absorb the iron owing to achlorhydria. In idiopathic hypochromic anæmia we probably have the same two causes tending to produce low vitamin $B_1$ in the blood as we have in the case of "alcoholic" polyneuritis, namely deficient intake and deficient absorption. But in the cases with anæmia, unlike the others, the intake is not markedly deficient and so the blood values do not fall so low. Possible deficiency of vitamin $B_1$, probably through failure of absorption, has been observed in other disorders of the gastro-intestinal canal, such as after gastro-enterostomy, entero-enterostomy, gastrectomy, in ulcerative colitis and in chronic bacillary dysentery.

Apart from failure to ingest or absorb the vitamin, deficiency is possible through its destruction in the gastro-intestinal canal. A number of cases of poly-neuritis secondary to pyloric stenosis have been described, and I have found low $B_1$ blood values in three such cases.[3] It seems probable that prolonged fermentation in the dilated stomach in an alkaline medium causes destruction of the vitamin, particularly since it is known to be unstable in presence of alkali. Indeed, it is possible that deficiency in the other cases of achlorhydria mentioned above may be due to actual destruction of the vitamin rather than to failure to absorb it. It should be mentioned, however, that Ungley's case[4] of polyneuritis secondary to pyloric stenosis had hyperchlorhydria. Of course the vitamin may be lost through vomiting, but in several cases described the neuritis preceded the vomiting.

### *Chemistry and Mode of Action of Vitamin $B_1$.*

The chemical work done in various laboratories led finally to the synthesis of vitamin $B_1$ by Williams and Cline in 1936, and also by others. The molecule contains a pyrimidine and a thiazole ring (Fig. 1).

FIG. 1.

Vitamin $B_1$ hydrochloride.

The way in which the vitamin acts in the body has been worked out mainly by Peters (see *e.g.* Peters,[5] 1936). Using pigeons and rats, he has shown that when vitamin $B_1$ is deficient, lactic and pyruvic acids accumulate in the body, because they are formed from carbohydrate but cannot be further broken down. Avitaminous brain tissue *in vitro* is unable to oxidize pyruvic acid, but does so when minute amounts of $B_1$ are added. Normal tissue oxidizes pyruvic acid and is unaffected by additional $B_1$. It therefore seems that vitamin $B_1$ is necessary for the oxidation of pyruvic acid in the body:

$$\text{Glucose} \longrightarrow \text{lactic acid} \xrightarrow{+O} \text{pyruvic acid} \xrightarrow[\uparrow \atop \text{vitamin } B_1]{+O} CO_2 + H_2O$$

This work on lower animals is confirmed by the study of patients with beriberi. Japanese workers showed that lactic acid accumulated in the body in this disease, and more recently Platt and Lu[6] (1937) have shown that the same is true of pyruvic acid. This biochemical change occurs particularly in the brain, and this goes far towards explaining the symptoms of beriberi. Pigeons which are moribund (blind and opisthotonic) from acute deficiency of vitamin $B_1$ recover completely within half an hour of the injection of the pure vitamin. The change producing these symptoms must obviously be biochemical, for no structural lesions can be demonstrated by ordinary histological methods of staining. It seems that the biochemical change in the nerve cells is sufficient to explain the peripheral neuritis of beriberi, because if the metabolism of the nerve cell is disturbed the neurone will be affected distally at first. It is not surprising, therefore, that the early symptoms occur as a result of changes at the distal end of the long nerves. The fact that the respiration of the auricle is also lowered in deficiency of vitamin $B_1$ helps to explain the cardiac manifestations.

Pure vitamin $B_1$ is now readily available for therapeutic use, either orally or parenterally. It is certain that there is no danger of overdosage or cumulative toxic effects, since a therapeutic dose of 5 mg. intravenously is about 1/7,200th of the toxic dose. Intravenous administration of as much as 100 mg. is without untoward effects. It would seem advisable to administer the vitamin by injection if there is doubt about its efficacy by mouth; for instance, Badger and Patrick found little effect on the neuritis of leprosy after oral administration, but good results after intramuscular injection. Treatment should be started with intramuscular or intravenous injection of from 20 to 50 mg. daily. Later the same dose may be given orally, or the injections decreased to 10 mg. daily until the patient is completely relieved of symptoms.

### VITAMIN $B_2$ COMPLEX.

It has already been stated that vitamin $B_2$ is now known to consist of at least four factors: riboflavin, vitamin $B_6$, filtrate factor and nicotinic acid.

Riboflavin, which has been synthesized and has the formula shown in figure 2, is necessary for growth in various animals and probably man. It prevents certain disorders in rats (such as conjunctivitis, alopecia, cataract* and even pediculosis), and

* It has recently been claimed that cataract is due to deficiency of vitamin $B_1$, since in human lenses with cataract the amount of this vitamin is very low and the amount of pyruvic acid is much increased.

dermatitis in turkeys. So far, it has no human significance. It is present in yeast, milk and liver, and is destroyed by light ; it will be further mentioned on p. 187.

FIG. 2.
Riboflavin.

Vitamin $B_6$ has recently been crystallized, but its formula is not yet known. In rats it prevents a symmetrical dermatitis which is called " rat acrodynia " because it is alleged to resemble pink disease in children. It may be mentioned that pink disease is believed by many to be a deficiency disease. There is as yet little or no evidence in favour of this view, and it seems rather more probable that the primary cause will prove to be a virus. This very interesting disease cannot be further discussed here ; it may be remarked, however, that in our present state of ignorance about the ætiology, it would be wise to administer a yeast preparation. Dried powdered brewers' yeast is a convenient source of the vitamin B complex ; about 30 g. daily is a reasonable dose for an adult. It is easily administered stirred into milk, or in warm water with salt.

Pellagra is very rarely recognized in this country,*

* An American colleague with considerable experience of pellagra in America recently saw a tramp with pellagrous dermatitis in a London bus.

although still common in some parts of America. The symptoms may be summarized as weakness and lassitude, anorexia, diarrhœa, sore ulcerated mouth and ulcerated atrophic tongue of a fiery red, mental changes and typical cutaneous lesions. These latter consist of a very characteristic dermatitis on the exposed surfaces, of lesions about the genitalia, seborrhœa on the face and neck, and hyperkeratoses over bony prominences (particularly the elbow). The story of the discovery of the P-P factor may briefly be summarized. Warburg and von Euler showed that nicotinic acid amide is the active group in two important coenzymes. This led to its use in other fields, and late last year Elvehjem and others showed that it cured " black-tongue "—a disease of dogs analogous to pellagra. Several groups of workers immediately tried it and certain related compounds in cases of pellagra, and there seems no doubt that the disease can be completely cured by the oral administration of nicotinic acid (Fig. 3) or its amide ; coramine, which is a related compound, is also effective (Spies *et al.*, 1938).[7] It is interesting to note that the peripheral neuritis of pellagra is not relieved by nicotinic acid, but is cured by vitamin $B_1$. Pellagra is occasionally seen in this country accompanying ulcerative colitis, and recently nicotinic acid has been tried in this latter condition. The acid is cheap, and the dose recommended by Spies is a total of 500 mg. a day, given by mouth in doses of 50 mg. The dose is carefully stated because it must be emphasized that, unlike vitamin $B_1$, nicotinic acid is definitely toxic, large amounts producing flushing, burning and itching of the skin, and increased motility of the stomach.

FIG. 3.
Nicotinic acid.

*Enzymes, Coenzymes and Phosphorylation.*

There is a striking connection between certain members of the vitamin B complex (and one or two other vitamins) and coenzymes or enzymes. An enzyme is an organic catalyst which is a protein ; a coenzyme is a non-protein organic compound which is fairly specific and associated with an enzyme in nature. Enzymes are believed to consist of an active (" prosthetic ") group, which is often unstable, joined to or part of a protein. If the prosthetic group is stable and becomes detached from its carrier, it may easily be mistaken for a coenzyme. It is probable that some coenzymes are nothing more than the active part of the enzymes. The composition of some coenzymes is known ; two which are important in cell respiration and fermentation contain nicotinic acid amide (see p. 185) combined through phosphate molecules with adenosine (a nucleoside). It has recently been shown by Lohmann that the coenzyme " cocarboxylase " (which catalyses the breakdown of pyruvic acid) is nothing else than vitamin $B_1$ combined with two phosphate molecules. Further, it is believed that riboflavin acts in the body combined with phosphate ; and a factor which was shown by Warburg to be important in cell respiration (and is sometimes called the "yellow enzyme") consists of riboflavin joined through phosphate to protein. Hence we have at least three members of the vitamin B complex ($B_1$, riboflavin and nicotinic acid amide) combined in the body with phosphate to form coenzymes or (if protein be attached) enzymes which are important in cell respiration or metabolism. By analogy with riboflavin it is tempting to believe that vitamin $B_1$ diphosphate is not merely the coenzyme which is necessary for the degradation of pyruvic acid, but is the active part of the enzyme which catalyses this degradation. In support of this hypothesis, I have recently found that neither vitamin $B_1$ nor the diphosphate exists free in blood, but that the vitamin is combined probably with protein.

Verzár[8] has stressed the importance of phosphorylation in the body. He has shown that fatty acids and certain carbohydrates are absorbed from the intestine at an increased rate by being combined with phosphate in the cells of the intestinal mucosa. For this process to occur the presence of riboflavin phosphate is necessary, and for riboflavin to be phosphorylated the hormone of the adrenal cortex is necessary. He maintains that adrenalectomized animals show a large decrease in the "yellow enzyme" in their livers, and can be caused to grow by being fed with riboflavin phosphate. Further, since he believes that fatty acids are not absorbed from the intestine unless phosphorylation can occur, he suggests that cases of idiopathic steatorrhœa, sprue and cœliac disease should be treated with the adrenal cortical hormone or with riboflavin phosphate. This work, however, is still in the experimental stage. But the possibility should be borne in mind that symptoms of deficiency of vitamin $B_1$ (or of other factors) may arise, not through actual deficiency of the vitamin, but through failure of it to be phosphorylated (and possibly combined with protein). Vitamin $B_1$ is phosphorylated in the liver (Ochoa and Peters) and possibly also in the intestine (Tauber). Apparent $B_1$ deficiency occurs occasionally in cases of cœliac disease and sprue, and has been described in liver disease.

*Anæmia and the vitamin B complex.*

A relation between the vitamin B complex and various forms of anæmia has been repeatedly claimed since 1929. Castle and Strauss (1932) stated that "vitamin $B_2$" or a closely related substance was the extrinsic factor in pernicious anæmia, but this was later discarded. More recently it has been shown that pigeons on a synthetic diet develop an anæmia which is cured by yeast but not by vitamins $B_1$ or $B_6$ or riboflavin. Clutterbuck, Evans and Wills (1937) consider that a factor related to the vitamin $B_2$ complex cures nutritional anæmia in monkeys ; it has been stated that the filtrate factor is concerned. György has produced aplastic anæmia with symptoms of hæmorrhagic diathesis in rats by dietary means, and cured it with nicotinic acid. Others have produced a severe microcytic hypochromic anæmia in puppies by a diet deficient in vitamin $B_6$. These results are difficult to correlate at present, but they indicate a possible relation between a factor or factors in the vitamin B complex and normal blood formation.

A short time ago vitamins were thought to have a narrow clinical application, being useful only in such classical diseases as rickets, scurvy, beriberi and pellagra. Now the pendulum has swung too far in the opposite direction and there is a danger of diseases not of nutritional origin being treated with vitamin preparations to the exclusion of appropriate therapy; there are instances of cardiovascular syphilis, diabetic neuritis and other conditions being diagnosed as deficiency of vitamin $B_1$ and treated with the pure vitamin. The rapid advance of laboratory work in this field makes the careful clinical investigation of each case even more important and emphasizes our ignorance of nutritional diseases in man. The relative ease with which several of such diseases can be treated with pure preparations is unfortunately apt to overshadow the important problem of preventing nutritional disorders by ensuring that everyone can consume a well-balanced diet.

### SUMMARY.

1. The various factors which compose the vitamin B complex are mentioned.

2. The clinical aspects of deficiency of vitamin $B_1$, and factors which may cause that deficiency, are described. The vitamin has been synthetized, and it is concerned in the body with the oxidation of pyruvic acid.

3. The four known factors which constitute the vitamin $B_2$ complex (riboflavin, vitamin $B_6$, filtrate factor and nicotinic acid) are briefly described. The symptoms and nature of pellagra are mentioned.

4. There is a striking connection between certain members of the vitamin B complex and coenzymes or enzymes. The importance of phosphorylation in the body is stressed.

5. A relation between deficiency of the vitamin B complex and anæmia is indicated.

### REFERENCES.

[1] Cowgill, G. R., *The Vitamin B Requirement of Man.* New Haven, 1934.

[2] Goodhart, R., and Jolliffe, N., *Jour. Amer. Med. Assoc.*, 1938, cx. 414.

[3] Laurent, L. P. E., and Sinclair, H. M., *Lancet*, 1938, i. 1045.

[4] Ungley, C. C., *Lancet*, 1938, i. 875 and 981.

[5] Petters, R. A., *Idem.*, 1936, i. 1161.

[6] Platt, B. S., and Lu, G. D., *Quart. Jour. Med.*, 1936, v. 355.

[7] Spies, T. D., and others, *Jour. Amer. Med. Assoc.*, 1938, cxi. 584.

[8] Verzár, F., and McDougall, E. J., *Absorption from the Intestine*, London, 1936.

### GENERAL REVIEWS.

Elvehjem, C. A., *Ergebn. Vitamin Hormonforsch.*, i. 140.

Grewe, R., *Ergebn. Physiol.*, 1937, xxxix. 252.

Peters, R. A., and O'Brien, J. R., *Ann. Rev. Biochem.*, 1938, vii. 305.

Strauss, M. B., *Jour. Amer. Med. Assoc.*, 1938, cx. 953.

Williams, R. R., *Ergebn. Vitamin Hormonforsch.*, i. 213.

## Figures and Tables

**Fig. 1. f1:**
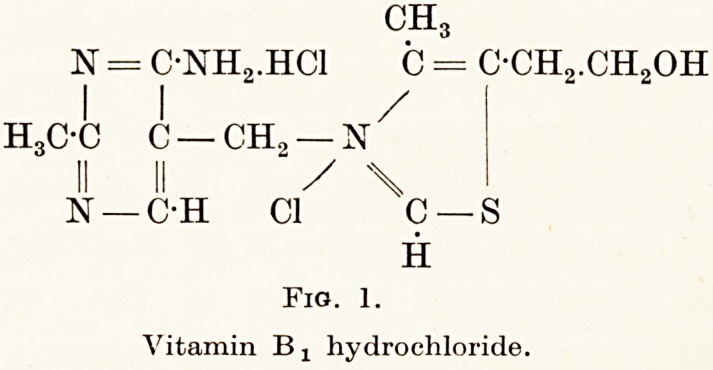


**Fig. 2. f2:**
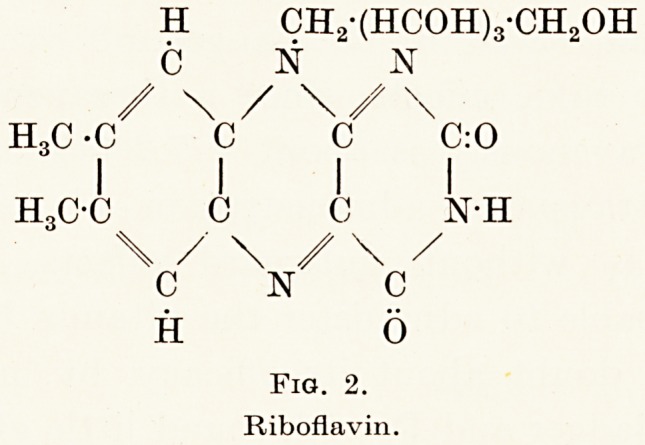


**Fig. 3. f3:**